# Molecular diagnosis of pediatric patients with citrin deficiency in China: *SLC25A13* mutation spectrum and the geographic distribution

**DOI:** 10.1038/srep29732

**Published:** 2016-07-11

**Authors:** Wei-Xia Lin, Han-Shi Zeng, Zhan-Hui Zhang, Man Mao, Qi-Qi Zheng, Shu-Tao Zhao, Ying Cheng, Feng-Ping Chen, Wang-Rong Wen, Yuan-Zong Song

**Affiliations:** 1 Department of Pediatrics, the First Affiliated Hospital, Jinan University, Guangzhou, 510630, China; 2 Clinical Medicine Research Institute, the First Affiliated Hospital, Jinan University, Guangzhou, 510630, China; 3 Department of Laboratory Science, the First Affiliated Hospital, Jinan University, Guangzhou, 510630, China

## Abstract

Citrin deficiency (CD) is a Mendelian disease due to biallelic mutations of *SLC25A13* gene. Neonatal intrahepatic cholestasis caused by citrin deficiency (NICCD) is the major pediatric CD phenotype, and its definite diagnosis relies on *SLC25A13* genetic analysis. China is a vast country with a huge population, but the *SLC25A13* genotypic features of CD patients in our country remains far from being well clarified. Via sophisticated molecular analysis, this study diagnosed 154 new CD patients in mainland China and identified 9 novel deleterious *SLC25A13* mutations, i.e. c.103A > G, [c.329 − 154_c.468 + 2352del2646; c.468 + 2392_c.468 + 2393ins23], c.493C > T, c.755 − 1G > C, c.845_c.848 + 1delG, c.933_c.933 + 1insGCAG, c.1381G > T, c.1452 + 1G > A and c.1706_1707delTA. Among the 274 CD patients diagnosed by our group thus far, 41 *SLC25A13* mutations/variations were detected. The 7 mutations c.775C > T, c.851_854del4, c.1078C > T, IVS11 + 1G > A, c.1364G > T, c.1399C > T and IVS16ins3kb demonstrated significantly different geographic distribution. Among the total 53 identified genotypes, only c.851_854del4/c.851_854del4 and c.851_854del4/c.1399C > T presented different geographic distribution. The northern population had a higher level of *SLC25A13* allelic heterogeneity than those in the south. These findings enriched the *SLC25A13* mutation spectrum and brought new insights into the geographic distribution of the variations and genotypes, providing reliable evidences for NICCD definite diagnosis and for the determination of relevant molecular targets in different Chinese areas.

Citrin deficiency (CD) is a Mendelian disease entity due to biallelic mutations of *SLC25A13* gene. With 18 exons and 17 introns, this gene is localized to chromosome 7q21.3, and encodes citrin, the liver-type mitochondrial aspartate/glutamate carrier isoform 2 (AGC2)^1^,^2^,^3^. Citrin has proven to be a dimeric protein with the molecular weight of 147.7 ± 1.7 kDa, and has a three-domain structure: a calcium-regulated N-terminal domain (residues 1–319, 36 kDa) with eight EF-hand motifs, a mitochondrial carrier domain (residues 320–612, 32 kDa) with six transmembrane helices, and a C-terminal domain (residues 613–675, 6 kDa) with an amphipathic helix being involved in the calcium-dependent regulation of the opening and closing of the AGC2 vestibule^4^.

Three age-dependent CD phenotypes have been described, which manifest in neonates or infants as neonatal intrahepatic cholestasis caused by citrin deficiency (NICCD, OMIM #605814)^5^,^6^,^7^,^8^,^9^,^10^, in older children as failure to thrive and dyslipidemia caused by citrin deficiency (FTTDCD)^4^,^11^,^12^,^13^,^14^,^15^, and in adolescents and adults, usually between the ages of 11 and 79 years, as adult-onset citrullinemia type II (CTLN2, OMIM #603471)^1^,^8^,^9^,^16^. After the NICCD period, some individuals might directly step into FTTDCD and some could develop CTLN2 in one or more decades^5^,^17^,^18^,^19^,^20^.

The *SLC25A13* genetic testing has been recognized as a reliable method for the definitive diagnosis of NICCD. However, about 10–15% of the *SLC25A13* mutations could not be detected by conventional DNA analysis^21^,^22^, and the identification of these obscure mutations constitutes a challenge against the definite diagnosis of NICCD^23^,^24^. Although about 200 Chinese NICCD patients have been diagnosed via *SLC25A13* analysis to date^22^,^25^,^26^,^27^, this number was rather limited, since China might have 85700 CD patients according to provisional epidemiological data^19^,^27^,^28^. Moreover, the *SLC25A13* mutations worldwide demonstrate remarkable heterogeneity^12^,^22^,^23^,^24^,^27^,^29^,^30^,^31^,^32^,^33^,^34^,^35^,^36^,^37^,^38^. China is a vast country with a huge population, but the *SLC25A13* genotypic features of CD patients, including the mutation spectrum and their geographic distribution, remains far from being well clarified in our country.

In this study, besides the conventional DNA analyses, cDNA cloning and in silico prediction as well as functional analysis were carried out to definitely diagnose pediatric CD patients. In addition, we investigated the geographic distribution of the *SLC25A13* mutations and genotypes, and evaluated the allelic heterogeneity in an attempt to provide reliable evidences for the determination of relevant molecular diagnostic targets in different geographic areas of China.

## Results

### New CD patients and the establishment of a large pediatric cohort

**Figure 1:**
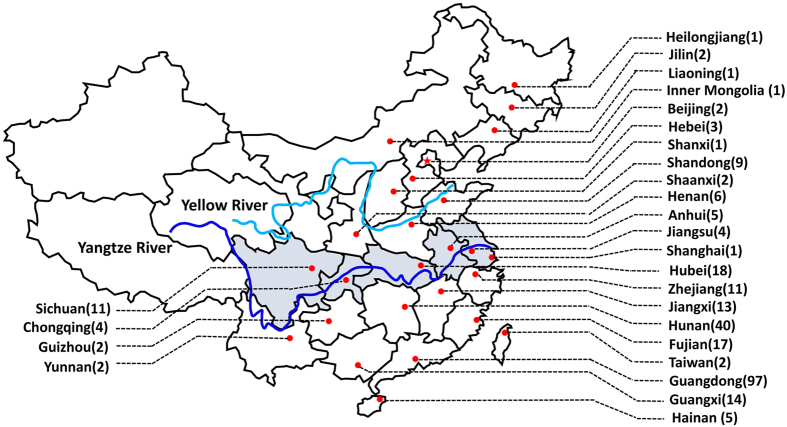
Native places of the 274 Chinese patients with citrin deficiency. By the end of February in 2016, 274 Chinese patients from 26 provinces, municipalities and autonomous regions in China were diagnosed. This figure was generated by means of the software WPS Office PowerPoint 2016, which was freely available at http://www.wps.cn/product/wps2016/. The base map was created by incrementally assembling the outlines of the Chinese administrative regions, which could be downloaded via the URL link http://www.pptstore.net/ppt_yuansu/12145.html, as a free network resource.

As shown in Table 1, this study diagnosed 154 new CD patients via sophisticated molecular, functional and bioinformatic analysis. Along with the 120 cases reported previously^23^,^24^,^27^,^33^,^34^, a large cohort with a total of 274 CD patients were established by our group from July, 2005 to the end of February, 2016. To the best of our knowledge, this is the largest CD cohort described in official references to date. This pediatric CD cohort encompassed 117 females and 157 males, and involved 264 families from 26 provinces, autonomous regions and municipalities in China (Fig. 1).

### Novel mutations and cDNA cloning analysis

**Figure 2:**
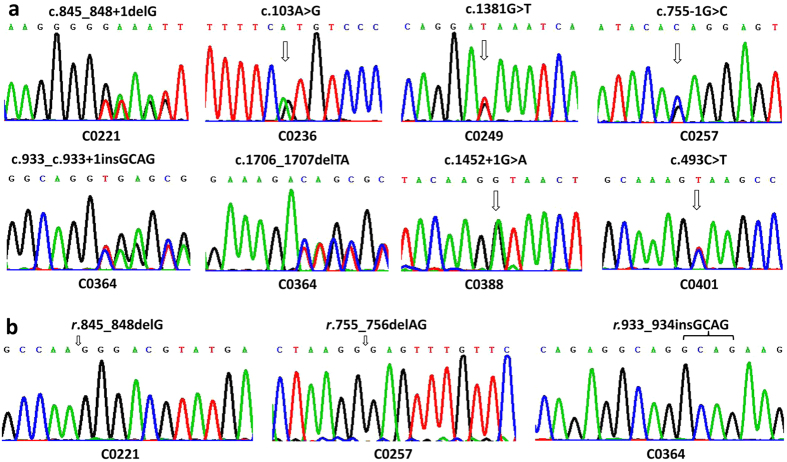
Novel mutations and ASVs identified by direct DNA sequencing analysis and cDNA analysis. The figure (**a**) showed the segmental DNA sequencing results of the 8 novel micro mutations. The figure (**b**) illustrated the cDNA sequencing results of the aberrant transcripts *r.*845_848delG, *r.*755_756delAG and *r.*933_934insGCAG, respectively, which were transcribed from the mutated alleles harboring c.845_c.848 + 1delG (C0221), c.755 − 1G > C (C0257) and c.933_c.933 + 1insGCAG (C0364), respectively.

As shown in Table 2, 9 novel *SLC25A13* mutations were identified in this study, and among them, c.103A > G (p.M35V), c.493C > T (p.Q165X), c.755−1G > C, c.845_c.848 + 1delG, c.933_c.933 + 1insGCAG, c.1381G > T (p.E461X), c.1452 + 1G > A and c.1706_1707delTA (p.S331fsX363) (Fig. 2a) were detected by Sanger sequencing of all the 18 *SLC25A13* exons and their flanking sequences. To address the issue whether and how c.755−1G > C, c.845_c.848 + 1delG and c.933_c.933 + 1insGCAG affect the splicing process of the relevant pre-mRNA molecules, cDNA cloning analysis of the transcripts from the affected *SLC25A13* alleles in PBLs were performed. They were found to give rise to the aberrant transcripts *r.*755_756delAG (p.252fs269X), *r.*845_848delG (p.D283fsX285) and *r.*933_934insGCAG (p.A312fsX317), respectively (Fig. 2b).

**Figure 3:**
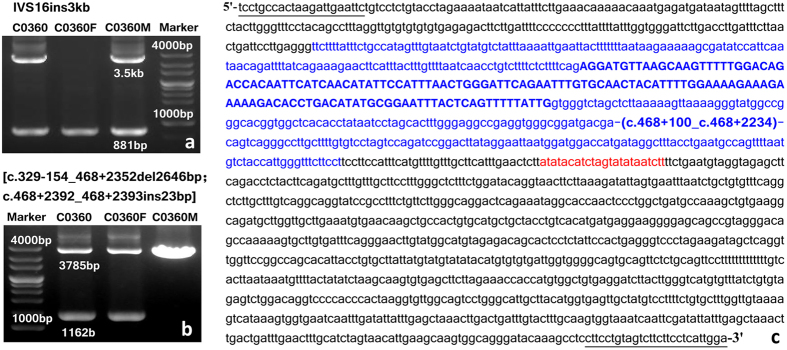
Identification of the mutation [c.329−154_468 + 2352del2646bp; c.468 + 2392_468 + 2393ins23bp] in patient C0360. (**a**) High-frequency mutation screening revealed the maternally-inherited mutation IVS16ins3kb. (**b**) Electrophoresis of the LA-PCR products with the primers covering exon 5 revealed an unexpected band of 1162 bp in size, which was inherited from the father. **(c)** Sanger sequencing of the 1162 bp product uncovered a 2646 bp deletion (sequences in blue), which spanned the entire exon 5 (capitals) and partial sequences of the introns 4 and 5. Meanwhile, a 23 bp insertion (sequences in red) which was 40nt behind the breakpoint was also discovered. Underlined were the primers for LA-PCR screening.

In patient C0360, four high-frequency mutation screening just detected the maternal mutation IVS16ins3kb. Further cDNA cloning analysis revealed that all the transcripts from the paternal allele featured exon 5 skipping (Table 3). The following screening of large insertion/deletion with primer set located in the adjacent intronic regions of exon 5 revealed a paternally-inherited unexpected PCR band of 1 kb in size, as illustrated in Fig. 3. Direct sequencing of this unexpected product revealed a 2646 bp deletion, involving the entire exon 5 and the adjacent intronic sequences, along with a 23 bp insertion 40 bp behind the breakpoint. According to the nomenclature rules^39^,^40^, this complex mutation was described as [c.329−154_c.468 + 2352del2646; c.468 + 2392_c.468 + 2393ins23], predictively leading to the production of a truncated citrin molecule p.E110fs127X.

In patient C0388, the maternal *SLC25A13* allele harbored two mutations [c.851_854del4; c.1452 + 1G > A], while the paternal mutation was not detected by direct DNA sequencing. The subsequent cDNA cloning analysis also shown that all the transcripts were from the maternal allele with *r.*851_854del4; moreover, half of them (12/21) demonstrated exon 14 skipping (*r*.1312_1452del, p.Ala438_Lys484del) (Table 3), indicating that the mutation c.1452 + 1G > A affected the splicing process of pre-mRNA molecules transcribed from the maternal allele.

### Bioinformatic and functional findings of the novel missense mutation c.103A > G (p.M35V)

Alignment analysis in 11 different species indicated that the amino acid M35 in citrin protein is highly conserved (Supplemental Information 1). The probability value of disease-causing potential was >0.9999 upon MutationTaster analysis and strongly indicated its deleterious nature. Meanwhile, this mutation is predicted to be probably damaging by using PolyPhen-2 with a score of 0.989 (sensitivity: 0.72; specificity: 0.97), and have a deleterious effect by using PROVEAN with a score of −3.25.

**Figure 4:**
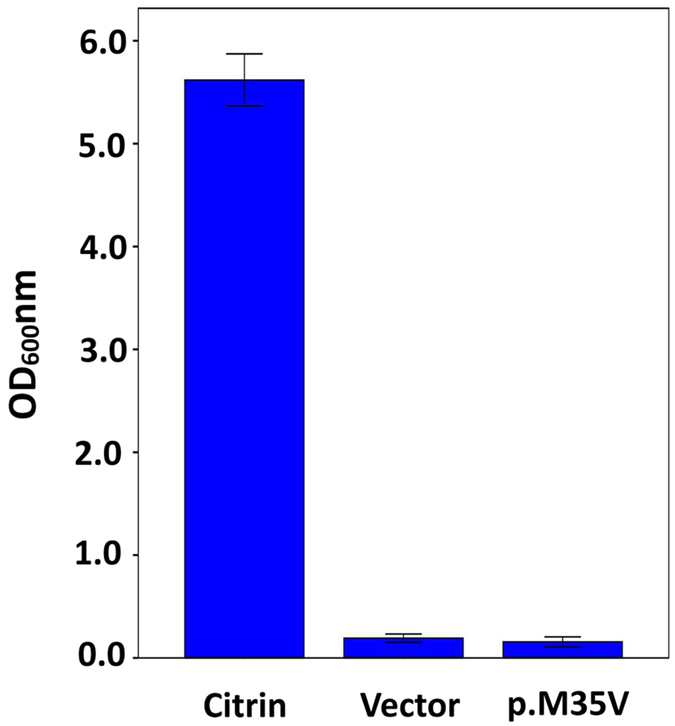
Functional analysis of the novel missense mutation c.103A > G (p.M35V). After growing for 96 hours, the growth ability of the yeast strain pYX212-mutant (p.M35V) was not significantly different (*P* = 0.341) from that transfected with the empty vector pYX212 (vector). However, both of them had significantly lower (*P* = 0.000) growth ability in comparison to pYX212-citrin (citrin), the yeast strain transfected with normal citrin recombinant. The results in each group were means ± SD of six repeated experiments.

As depicted in Fig. 4, after growing for 96 hours, the growth ability of the pYX212-mutant (p.M35V) was not significantly different (*P* = 0.341) from that of the empty vector pYX212 (vector). However, both of them had significantly lower (*P* = 0.000) growth ability in comparison to pYX212-citrin (citrin). These findings indicated that the mutation p.M35V, as a lack-of-function variation, caused the elimination of the AGC2 function of citrin protein.

### *SLC25A13* mutation spectrum and regional distribution

*SLC25A13* mutations/variations were detected in 522 out of the 528 independent alleles in Table 2, with the diagnostic efficiency of 98.86%. The *SLC25A13* spectrum was composed of 41 mutations/variations, including 12 missense mutations, 8 deletion, 10 nonsense, 4 splice-site, 3 insertion, 1 duplication, 1 pathogenic SNP^29^,^41^,^42^, 1 aberrant transcript^42^ and 1 complex mutation. The variations c.851_854del4 (58.33%), c.1638_1660dup (8.52%), IVS6 + 5G > A (7.58%) and IVS16ins3kb (10.04%) constituted the high-frequency mutations on top of the list. Three other mutations, IVS4ins6kb, IVS11 + 1G > A and c.1399C > T (p.R467X), were found at relative frequencies of 1–2%, and the remaining 34 mutations each had frequencies <1%.

Among all the 41 mutations/variations, as shown in Table 2, 16 ones were detected in the north, 16 in the border and 27 in the south. Meanwhile, there were 6 private mutations in the north, 8 in the border and 15 in the south. The comparisons of their relative frequencies among the three areas revealed that c.775C > T (p.Q259X), c.851_854del4, c.1078C > T (p.R360X), IVS11 + 1G > A, c.1364G > T (p.R455L), c.1399C > T (p.R467X) and IVS16ins3kb presented with significantly different geographic distribution with *P* all <0.05 (Table 2). In pairwise comparisons, the southern population had a higher relative frequency of c.851_854del4 than the border (*P *=* *0.001) and northern (*P *=* *0.000) populations. By contrast, the relative frequency of IVS16ins3kb was lower in the south than in the border (*P *=* *0.013) and the north (*P *=* *0.005). Besides, when compared with the south region, the relative frequencies of c.1399C > T (p.R467X) (*P *=* *0.003) in the border and those of c.1364G > T (p.R455L) (*P *=* *0.012) and IVS11 + 1G > A (*P *=* *0.015) in the north were higher. No significant difference were observed on the geographic distribution of the mutations IVS6 + 5G > A and c.1638_1660dup among different areas.

### *SLC25A13* genotype and allelic heterogeneity

Except the 6 patients with only one mutation detected and the 13 ones with parents of different geographic origins, in this paper, the remaining 245 unrelated individuals were enrolled for the comparison of the genotype distribution. As shown in Table 4, there were 23 individuals from the north, 35 from the border and 187 from the south, respectively. Among a total of 53 genotypes, the four genotypes c.851_854del4/c.851_854del4, c.851_854del4/IVS16ins3kb, c.851_854del4/IVS6 + 5G > A and c.851_854del4/c.1638_1660dup were dominant with the relative frequencies of 42.04%, 10.61%, 7.76% and 6.94%, respectively. Despite of the marked diversity of the genotypes, only c.851_854del4/c.851_854del4 and c.851_854del4/c.1399C > T (p.R467X) demonstrated significantly different geographic distribution with *P* < 0.05. Further pairwise comparisons revealed that the relative frequency of c.851_854del4/ c.851_854del4 in the south was much higher than that in the north (*P* = 0.000).

The observed homozygosity calculated based on the genotype frequencies was 17.39% (4/23) in the north, 34.28% (12/35) in the border and 52.94% (99/187) in the south, respectively. In addition, the theoretical homozygosity value, which was calculated based on allele frequencies, was 15.24% in the north, 24.31% in the border and 44.81% in the south, respectively. In pairwise comparisons, both of the observed and theoretical homozygosity values were higher in the south than in the north, with the *P* values of 0.001 and 0.012, respectively. In other words, the northern population had a higher level of allelic heterogeneity at the *SLC25A13* locus than the southern population.

## Discussion

The first case of NICCD in mainland China was reported by our group in 2006^43^. Since then, more and more Chinese CD patients were definitely diagnosed by *SLC25A13* genetic analysis in our department^23^,^24^,^27^,^33^,^34^. During this process, conventional DNA analytic approaches such as PCR/LA-PCR, PCR-RFLP and Sanger sequencing played important roles. In this study, as shown in Table 2, the four mutations c.851_854del4, c.1638_1660dup, IVS6 + 5G > A and IVS16ins3kb together had a relative frequency of 84.47%, indicating that the screening of these high-frequency mutations should be initially performed for the rapid molecular diagnosis of CD patients. In addition, direct sequencing of the 18 *SLC25A13* exons and their adjacent intronic regions could identified the remaining micro mutations, which accounted for 12.5%. Besides, the functional and bioinformatic tools also made substantial contribution to the pathogenicity confirmation of the novel missense mutation c.103A > G (p.M35V). As a result, the 154 new CD patients diagnosed in this paper, together with those reported in our department previously, constituted a 274-case cohort. So far as we know, this is the hitherto largest CD cohort in official references worldwide, laying a foundation for our subsequent clinical investigation. In particular, the 9 novel mutations identified in this study enriched the *SLC25A13* mutation spectrum, and provided reliable laboratory evidences not only for the definite diagnosis of the corresponding individuals, but also for the genetic counseling of their families in the future.

It was noteworthy that, as an unique technique developed by our group^42^, the *SLC25A13* cDNA cloning analysis using PBLs had proved to be a feasible tool for the detection of the large insertion/deletion mutations^23^,^24^,^27^. In this paper, this molecular tool also played unique roles in the definite diagnosis of NICCD patients. The aberrant transcripts detected by cDNA analysis proved that c.755 − 1G > C, c.845_c.848 + 1delG, c.933_c.933 + 1insGCAG and c.1452 + 1G > A all had influence on the splicing process of the pre-mRNA. In addition, by using this tool, the novel mutation [c.329 − 154_c.468 + 2352del2646; c.468 + 2392_c.468 + 2393ins23] in patient C0360 was identified as the third large mutation resulting in exon 5 skipping (*r.*329_468, p.E110fs127X) following the mutation IVS4ins6kb (GenBank accession number: KF425758)^27^ and c.329 − 1687_c.468 + 3865del^24^. Of particular note, in patient C0388, we confirmed that the maternally-inherited allele harbored two deleterious mutations based on the cDNA cloning results. Although the paternal mutation remained to be explored currently, the undetectable transcriptional product from the paternal allele clearly indicated the existence of a pathogenic mutation, providing direct laboratory evidences supporting the CD diagnosis. Unfortunately, due to the lack of fresh PBLs or liver specimens for further cDNA cloning analysis, mutations in 6 *SLC25A13* alleles remained obscure in this study; however, the rate of unidentified mutations (1.14% of all mutated alleles) in our study is much lower than those in previous publications using the conventional molecular approaches only. *SLC25A13* cDNA cloning analysis using PBLs should be taken as an important tool for the molecular diagnosis of CD patients.

Although the relative frequencies of c.1638_1660dup and IVS6 + 5G > A were uniform in different regions of China, some *SLC25A13* mutations demonstrated different geographic distribution in this study. The relative frequencies of c.851_854del4 tended to decrease gradually from south to north, while that of IVS16ins3kb had an opposite tendency, as shown in the Table 2. The genetic variation following a continuous pattern from south to north might be attributed to the genetic flow occurred between distinct populations. Recent genetic studies have suggested that modern humans colonized East Asia via Southern and Northern routes on both sides of the Himalayas. Genetic flow between populations, which took place when the two migration routes overlapped, probably lasted a long time, resulting in the continuous pattern of genetic variation^44^,^45^. The continuous patterns of genetic variation at the *SLC25A13* locus in the present study are compatible with this presumed migration model. Actually, previous haplotype study^28^ suggested that c.851_854del4 originated around the Guangxi and Yunan areas. Its higher frequency in south China can be explained as a result of the founder effect, while its lower frequency in the north, by genetic drift. Interestingly, besides the four common mutations, c.1399C > T (p.R467X) was relatively common in the border, while IVS11 + 1G > A and c.1364G > T (p.R455L) were relatively common in the north. This phenomenon might be attributed to different founding populations but a lower migration rate among different areas in mainland China. These mutations should be considered as targets when establishing a screening strategy for CD patients in relevant populations.

Moreover, although a diversity of genotypes with a total number of 53 was discovered in the large CD cohort, c.851_854del4/c.851_854del4 was the unique genotype with higher relative frequency in the south than in the north (49.20% *vs* 4.35%), as shown in Table 4. This could be explained once again by the aforementioned founder effect of the c.851_854del4 mutation, which might occurred in a far remote ancestor in the south; Furthermore, subsequent homozygosity comparison demonstrated that, different from the CD patients from the south who had higher homozygosity, patients in the north showed higher allelic heterogeneity at the *SLC25A13* locus. This finding suggested that some CD patients might be missed while the CD prevalence be underestimated in the north, when the same high-frequency mutations as in the south were choosed as the molecular targets for the detection of CD patients and *SLC25A13* carriers in the north area. Therefore, the exploration of additional *SLC25A13* mutations should be regarded as an important issue in the north area in terms of CD molecular diagnosis and epidemic survey.

In summary, via sophisticated molecular, functional and in silico analysis of *SLC25A13* gene and its cDNA, this paper reported 154 new CD patients and identified 9 novel pathogenic mutations. The *SLC25A13* mutation spectrum in the hitherto largest CD cohort of 274 cases and their different geographic distribution formed a substantial contribution to the in-depth understanding of the genotypic feature of CD patients in China, and provided reliable evidences for the development of molecular diagnostic strategies in different Chinese areas.

## Methods

### Subjects and Ethics

This research enrolled a total of 274 CD patients diagnosed by our group in the past over 10 years, including 154 new CD cases which were diagnosed by sophisticated molecular, functional and bioinformatic analysis of *SLC25A13* gene and its cDNA, as described below, from February, 2013 to the end of February, 2016. Our study adheres to the ethical guidelines of the World Medical Association Declaration of Helsinki (WMADH 2008) and was approved by the Medical Ethical Committee of the First Affiliated Hospital, Jinan University. *SLC25A13* analyses were conducted with the written informed consents from the guardians of the patients.

### *SLC25A13* Mutation Analysis

Genomic DNA of the patients suspected to have CD and their parents was extracted from EDTA-anticoagulant peripheral venous blood. The 4 high-frequency mutations c.851_854del4, c.1638_1660dup, IVS6 + 5G > A and IVS16ins3kb were initially screened by using PCR/LA-PCR and PCR-RFLP, respectively. All the 18 *SLC25A13* exons and their adjacent intronic regions were amplified by PCR and analyzed by Sanger sequencing in patients with just one mutation was detected. Following that, if there was a *SLC25A13* mutation remained obscure, IVS4ins6kb, another large insertion with relative high frequency in Chinese, would be screened by LA-PCR as in our previous publications^27^.

### Reverse transcription-PCR (RT-PCR) and cDNA cloning analysis

RT-PCR and cDNA cloning analysis were subsequently carried out in patients still with undetected mutation by all the approaches above, and in those with novel mutations that might affect the splicing of pre-mRNA molecules. In brief, total RNA was extracted from peripheral blood lymphocytes (PBLs), which were collected from 2 ml fresh EDTA-anticoagulant peripheral venous blood. Then RT-PCR was performed to synthesized cDNA following the kit manufacture’s protocol (Invitrogen, USA). With the cDNA as template, nest-PCR was then performed for the target products, and the purified nest-PCR products were cloned into PMD-18T vector (Takara, Japan) and transformed into DH5α Escherichia coli competent cells. The positive clones were tested by LA-PCR with the universal primer set RVM (GAGCGGATAACAATTTCACACAGG) and M13-47 (CAGCACTGACCCTTTTGGGACCGC) and then sequenced, as previously described^42^.

### *In silico* analyses

The conservative property of the amino acid affected by the novel missense mutation was analyzed by the software Clustal Omega (http://www.ebi.ac.uk/Tools/msa/clustalo/)^46^. The amino acid sequences of human citrin were comparatively aligned with other homologous proteins from 10 different eukaryotic species, including chimpanzee, mouse, rat, dog, cow, pig, opossum, chicken, xenopus tropicalis and caenorhabditis elegans. All of these amino acid sequences were obtained from the NCBI database (www.ncbi.nlm.nih.gov). Then the pathogenicity of the missense mutation was predicted by the softwares PolyPhen-2 (http://genetics.bwh.harvard.edu/pph2/)^47^,^48^, mutationTaster (http://mutationtaster.org/ MutationTaster/index.html)^49^ and PROVEAN (http://provean.jcvi.org/index.php)^50^,^51^, respectively.

### Functional study

A diploid AGC1-disrupted yeast model, BYagc1Δ, which was constructed in our previous publication^34^, was used to evaluate the functional effect of the novel missense mutation. The normal citrin-coding sequence (NM_014251.2) was amplified and the novel missense mutation was introduced into the wild type *SLC25A13* cDNA by overlap-extension PCR. These products were purified and cloned into the vector pYX212 (Novagen, USA) to constitute the plasmid pYX212-citrin and pYX212-mutant, respectively. The BYagc1Δ strains were then transfected with the recombinant plasmids and the empty vector pYX212, respectively. The positive clones were screened using the uracil minus medium SD-URA and cultured in SA medium with acetate as the unique carbon source. After 96 hour of culture, the growth abilities of these three strains were assessed by the cell density measured at OD_600_.

### Geographic division

The Yangtze River has been considered as a historically significant boundary of the Chinese population^52^,^53^,^54^. In addition, the previously estimated carrier rate of *SLC25A13* mutations was 1/48 in the south but 1/940 in the north of this river^28^. Accordingly, the distribution of the mutations and genotypes in this study were compared among the north, border and south regions relevant to this boundary, based on the origin of the parents of each case. In this study, individuals in the north area referred to those from Beijing, Inner Mongolia, Shangdong, Shanxi, Shaanxi, Henan, Hebei, Liaoning, Jilin and Heilongjiang; in the border, from Shanghai, Jiangsu, Anhui, Hubei, Sichuan and Chongqing; and in the south, form Guangdong, Guangxi, Yunnan, Guizhou, Hunan, Fujian, Zhejiang, Jiangxi, Hainan and Taiwan, respectively.

### Calculation of homozygosity

The theoretical homozygosity (J) at a locus in a given population is measured by J = ∑*Χ*_*i*_^2^, where ∑ stands for summation over all alleles, and *Χ*_*i*_ is the frequency of the *i*th allele^55^,^56^. If the number of the *i*th allele is m, *Χ*_*i*_ is calculated to be m/N, where N is the total number of the mutant alleles being investigated. The alleles harboring obscure mutations were counted as *SLC25A13* alleles different from those with detected variations, and thus, each of them was defined to have a frequency of 1/N.

### Statistical analysis

The frequencies of the *SLC25A13* mutations and genotypes, as well as the homozygosity values among the three geographic areas, were compared by means of Chi-square test or Fisher’s exact tests, respectively. When the Chi-square test of 3 × 2 table was significant with *P* < 0.05, pairwise comparisons were then performed with Bonferroni corrections of the *P* values. There were 3 pairwise comparisons: (1) north *vs* border, (2) north *vs* south, and (3) border *vs* south; accordingly, the adjusted *P* values of 2 × 2 Chi-square test for significance was 0.017 (0.05/3)^57^. The data of growth abilities of the yeast strains were analyzed by using one-way ANOVA followed by the Games-Howell test for the pairwise comparison of the non-homogeneity of variances, with *P* < 0.05 as the significant criteria. All statistical calculations were performed on the software SPSS17.0.

## Additional Information

**How to cite this article**: Lin, W.-X. *et al*. Molecular diagnosis of pediatric patients with citrin deficiency in China: *SLC25A13* mutation spectrum and the geographic distribution. *Sci. Rep.* **6**, 29732; doi: 10.1038/srep29732 (2016).

## Supplementary Material

Supplementary Information

## Figures and Tables

**Table 1:** Molecular diagnosis of 154 new NICCD patients.

No.	Patients	Gender	*SLC25A13* mutations	No.	Patients	Gender	*SLC25A13*mutations
01	C0171	Male	c.851_854del4/ c.851_854del4	78	C0289	Male	IVS16ins3kb/ IVS16ins3kb
02	C0172	Male	c.851_854del4/c.1064G > A	79	C0290	Male	c.851_854del4/ c.851_854del4
03	C0173	Female	c.550C > T(p.R184X)/IVS4ins6kb	80	C0291	Female	c.550C > T(p.R184X)/ c.1638_1660dup
04	C0174	Female	c.851_854del4/ c.851_854del4	81	C0293	Male	IVS6 + 5G > A/ IVS16ins3kb
05	C0175	Female	c.495delA/IVS16ins3kb	82	C0294	Female	c.851_854del4/ c.851_854del4
06	C0176	Male	c.851_854del4/ c.851_854del4	83	C0297	Male	c.1092_1095delT/ c.851_854del4
07	C0177	Male	c.851_854del4/ c.851_854del4	84	C0298	Male	c.851_854del4/IVS16ins3kb
08	C0178	Female	c.851_854del4/ c.851_854del4	85	C0298S	Female	c.851_854del4/IVS16ins3kb
09	C0180	Female	c.1638_1660dup/IVS16ins3kb	86	C0299	Female	c.851_854del4/IVS16ins3kb
10	C0181	Male	c.2T > C/?	87	C0301	Male	c.851_854del4/ c.1638_1660dup
11	C0182	Female	c.851_854del4/ c.851_854del4	88	C0303	Male	c.851_854del4/c.1048G > A(p.D350N)
12	C0183	Female	c.851_854del4/IVS16ins3kb	89	C0306	Female	c.851_854del4/ IVS6 + 5G > A
13	C0184	Female	c.851_854del4/IVS4ins6kb	90	C0307	Female	c.851_854del4/ c.851_854del4
14	C0185	Male	c.851_854del4/ c.851_854del4	91	C0310	Male	c.851_854del4/ c.851_854del4
15	C0189	Male	c.851_854del4/ IVS6 + 5G > A	92	C0314	Female	c.851_854del4/ c.851_854del4
16	C0190	Female	c.851_854del4/ c.851_854del4	93	C0315	Male	c.851_854del4/ c.851_854del4
17	C0191	Female	c.851_854del4/ c.851_854del4	94	C0316	Female	c.1638_1660dup/IVS16ins3kb
18	C0192	Female	c.851_854del4/IVS16ins3kb	95	C0319	Male	c.851_854del4/ c.851_854del4
19	C0197	Female	c.851_854del4/IVS6 + 5G > A	96	C0320	Male	c.851_854del4/ c.851_854del4
20	C0201	Male	c.851_854del4/ c.851_854del4	97	C0321	Male	c.1638_1660dup/IVS6 + 5G > A
21	C0205	Male	c.851_854del4/ c.851_854del4	98	C0323	Male	c.851_854del4/ c.851_854del4
22	C0206	Male	c.851_854del4/ c.851_854del4	99	C0324	Male	c.851_854del4/ c.851_854del4
23	C0207	Male	c.851_854del4/ IVS16ins3kb	100	C0325	Female	c.851_854del4/ c.851_854del4
24	C0208	Male	c.851_854del4/c.775C > T(p.Q259X)	101	C0327	Male	c.851_854del4/ c.851_854del4
25	C0209	Male	c.851_854del4/ c.1638_1660dup	102	C0335	Female	c.851_854del4/ c.1638_1660dup
26	C0211	Female	c.851_854del4/ c.851_854del4	103	C0336	Male	c.851_854del4/c.955C > T(p.R319X)
27	C0212	Female	c.851_854del4/ c.851_854del4	104	C0337	Male	c.851_854del4/IVS16ins3kb
28	C0215	Female	c.851_854del4/ c.1638_1660dup	105	C0338	Female	c.851_854del4/IVS16ins3kb
29	C0216	Male	c.851_854del4/ c.851_854del4	106	C0339	Female	c.851_854del4/IVS16ins3kb
30	C0217	Male	c.851_854del4/ c.1638_1660dup	107	C0340	Female	c.851_854del4/ c.851_854del4
31	C0219	Female	c.851_854del4/ c.851_854del4	108	C0343	Male	c.851_854del4/ c.851_854del4
32	C0220	Female	c.851_854del4/IVS4ins6kb	109	C0344	Female	c.851_854del4/ c.851_854del4
33	C0221	Male	IVS11 + 1G > A/c.845_c.848 + 1delG	110	C0345	Female	c.851_854del4/ c.851_854del4
34	C0222	Female	c.851_854del4/IVS6 + 5G > A	111	C0347	Female	c.1048G > A(p.D350N)/IVS16ins3kb
35	C0224	Female	c.851_854del4/ c.851_854del4	112	C0348	Male	c.851_854del4/ c.851_854del4
36	C0226	Male	c.851_854del4/ c.1078C > T(p.R360X)	113	C0349	Male	c.851_854del4/IVS16ins3kb
37	C0227	Male	c.851_854del4/ IVS16ins3kb	114	C0350	Female	c.851_854del4/IVS6 + 5G > A
38	C0229	Male	c.851_854del4/ c.1638_1660dup	115	C0351	Male	c.851_854del4/ c.851_854del4
39	C0234	Female	c.851_854del4/ c.851_854del4	116	C0352	Male	c.851_854del4/ c.851_854del4
40	C0235	Female	c.851_854del4/ c.1638_1660dup	117	C0354	Male	c.1638_1660dup/IVS6 + 5G > A
41	C0236	Male	c.851_854del4/ c.103A > G(p.M35V)	118	C0355	Male	IVS11 + 1G > A/ c.1638_1660dup
42	C0237	Male	c.851_854del4/c.1078C > T(p.R360X)	119	C0356	Female	IVS6 + 5G > A/c.1399C > T(p.R467X)
43	C0238	Male	c.851_854del4/ c.851_854del4	120	C0357	Female	c.851_854del4/ c.851_854del4
44	C0239	Female	c.851_854del4/ c.851_854del4	121	C0358	Female	c.851_854del4/ IVS6 + 5G > A
45	C0240	Male	IVS6 + 5G > A/ IVS11 + 1G > A	122	C0359	Male	c.851_854del4/IVS16ins3kb
46	C0241	Male	c.1063C > G(p.R355G)/ IVS16ins3kb	123	C0360	Male	IVS16ins3kb/[c.329−154_c.468 + 2352del2646; c.468 + 2392_c.468 + 2393ins23]
47	C0242	Female	c.851_854del4/ c.851_854del4	124	C0361	Male	c.851_854del4/ c.851_854del4
48	C0243	Female	c.1638_1660dup/c.1638_1660dup	125	C0363	Male	c.1638_1660dup/IVS6 + 5G > A
49	C0244	Male	IVS16ins3kb/IVS16ins3kb	126	C0364	Male	c.933 + 1_c.933 + 2insCAGG/c.1706_1707delTA
50	C0245	Female	c.851_854del4/ c.851_854del4	127	C0366	Male	IVS16ins3kb/ IVS16ins3kb
51	C0246	Male	c.851_854del4/ c.851_854del4	128	C0367	Male	c.851_854del4/c.1048G > A(p.D350N)
52	C0247	Male	c.851_854del4/ c.851_854del4	129	C0368	Female	c.851_854del4/c.1638_1660dup
53	C0248	Female	c.851_854del4/IVS16ins3kb	130	C0369	Male	c.851_854del4/c.550C > T(p.R184X)
54	C0249	Female	c.1638_1660dup/c.1381G > T(p.E461X)	131	C0371	Female	c.851_854del4/c.1399C > T
55	C0250	Male	c.851_854del4/ c.851_854del4	132	C0373	Male	c.851_854del4/IVS16ins3kb
56	C0251	Female	IVS11 + 1G > A/ c.1638_1660dup	133	C0373S	Female	c.851_854del4/IVS16ins3kb
57	C0253	Female	c.1638_1660dup/IVS16ins3kb	134	C0375	Female	IVS16ins3kb/ c.851_854del4
58	C0253S	Female	c.1638_1660dup/IVS16ins3kb	135	C0376	Male	c.851_854del4/ c.851_854del4
59	C0257	Male	IVS7−1G > C/c.1364G > T(p.R455L)	136	C0377	Male	c.851_854del4/ c.851_854del4
60	C0258	Female	c.1638_1660dup/c.1638_1660dup	137	C0378	Male	c.851_854del4/ c.851_854del4
61	C0261	Male	c.851_854del4/ c.851_854del4	138	C0380	Male	c.851_854del4/ c.851_854del4
62	C0262	Male	c.851_854del4/ c.851_854del4	139	C0382	Male	c.851_854del4/IVS4ins6kb
63	C0264	Female	c.851_854del4/ c.1399C > T (p. R467X)	140	C0383	Female	c.1638_1660dup/c.1638_1660dup
64	C0266	Male	c.851_854del4/IVS6 + 5G > A	141	C0384	Male	c.851_854del4/ IVS16ins3kb
65	C0267	Female	c.851_854del4/IVS6 + 5G > A	142	C0385	Male	c.851_854del4/ IVS16ins3kb
66	C0268	Male	c.851_854del4/ c.1399C > T (p. R467X)	143	C0387	Male	IVS6 + 5G > A/IVS4ins6kb
67	C0270	Male	c.851_854del4/ c.851_854del4	144	C0388	Female	[c.851_854del4; c.1452 + 1G > A]/?
68	C0272	Female	c.851_854del4/ c.851_854del4	145	C0392	Female	c.851_854del4/ c.851_854del4
69	C0273	Male	c.851_854del4/ c.851_854del4	146	C0394	Male	c.1638_1660dup/IVS16ins3kb
70	C0276	Male	c.851_854del4/ c.851_854del4	147	C0395	Male	c.851_854del4/ c.1638_1660dup
71	C0277	Male	c.851_854del4/ c.851_854del4	148	C0396	Male	IVS6 + 5G > A/IVS6 + 5G > A
72	C0278	Female	c.851_854del4/ c.851_854del4	149	C0397	Female	c.851_854del4/ IVS6 + 5G > A
73	C0280	Female	c.851_854del4/ c.851_854del4	150	C0398	Female	c.851_854del4/ c.851_854del4
74	C0283	Female	c.851_854del4/ c.851_854del4	151	C0400	Male	c.851_854del4/ IVS6 + 5G > A
75	C0284	Male	IVS16ins3kb/IVS6 + 5G > A	152	C0401	Female	c.493C > T/ IVS16ins3kb
76	C0286	Male	IVS16ins3kb/ IVS16ins3kb	153	C0403	Female	c.851_854del4/ c.851_854del4
77	C0288	Male	c.851_854del4/IVS6 + 5G > A	154	C0404	Male	IVS4ins6kb/ c.851_854del4

**Table 2:** Spectrum of the *SLC25A13* mutation/variations identified in the CD patients and their relative frequency.

No.	Location	Systematic name (DNA level)	Amino acids	Types	Relative frequency (%)				*P*
					North(n = 55)	Border(n = 81)	South(n = 392)	China(n = 528)	
01	Ex1	c.2T > C	p.Met1_Phe34del	Pathogenic SNP※	0.00	0.00	0.77	0.57	1.000
02	Ex2_3	*r*.16-212dup	Unclear	Aberrant transcript▲	0.00	0.00	0.26	0.19	1.000
03	Ex3	c.72T > A	p.Y24X	Nonsense	0.00	1.23	0.00	0.19	0.258
04	Ex3	c.103A > G*	p.M35V	Missense	0.00	0.00	0.26	0.19	1.000
05	Ex4	c.265delG	p.D89fs94X	Deletion	0.00	0.00	0.26	0.19	1.000
06	Ex5	c.329−1687_c.468 + 3865del	p.E110fs127X	Deletion	0.00	0.00	0.26	0.19	1.000
07	Ex5	[c.329−154_c.468 + 2352del2646; c.468 + 2392_c.468 + 2393ins23]*	p.E110fs127X	Complex	0.00	1.23	0.00	0.19	0.258
08	Ex5	IVS4ins6kb	p.E110fs127X	Insertion	1.82	2.47	1.28	1.52	0.310
09	Ex6	c.475C > T	p.Q159X	Nonsense	0.00	1.23	0.00	0.19	0.258
10	Ex6	c.493C > T*	p.Q165X	Nonsense	0.00	1.23	0.00	0.19	0.258
11	Ex6	c.495delA	p.Q165fs195X	Deletion	0.00	1.23	0.00	0.19	0.258
12	Ex6	c.550C > T	p.R184X	Nonsense	0.00	1.23	0.51	0.57	0.592
13	IVS6	IVS6 + 5G > A	P.V260fs212X	Splice-site	3.64	8.64	7.91	7.58	0.494
14	Ex7	c.754G > A	p.E252K	Splice-site	1.82	0.00	0.26	0.38	0.221
15	IVS7	c.755−1G > C*	p.252fs269X	Deletion	1.82	0.00	0.00	0.19	0.104
16	Ex8	c.775C > T	p.Q259X	Nonsense	0.00	2.47	0.00	0.38	0.034
17	Ex8	c.790G > A	p.V264I	Missense	0.00	0.00	0.26	0.19	1.000
18	Ex8	c.847G > T	p.G283X	Nonsense	0.00	0.00	0.26	0.19	1.000
19	Ex8	c.845_c.848 + 1delG*	p.D283fsX285	Deletion	0.00	0.00	0.26	0.19	1.000
20	Ex9	c.851_854del4	p.R284fs286X	Deletion	27.27	44.44	65.56	58.33	0.000
21	Ex9	c.933_c.933 + 1insGCAG*	p.A312fsX317	Insertion	1.82	0.00	0.00	0.19	0.104
22	Ex10	c.955C > T	p.R319X	Nonsense	3.64	0.00	0.51	0.76	0.090
23	Ex10	c.998G > A	p.G333D	Missense	0.00	0.00	0.26	0.19	1.000
24	Ex11	c.1019_1177 + 893del	p.340_392del	Deletion	1.82	0.00	0.00	0.19	0.104
25	Ex11	c.1048G > A	p.D350N	Missense	0.00	0.00	1.02	0.76	1.000
26	Ex11	c.1063C > G	p.R355G	Missense	0.00	0.00	0.51	0.38	1.000
27	Ex11	c.1064G > A	p.R355Q	Missense	0.00	0.00	0.51	0.38	1.000
28	Ex11	c.1078C > T	p.R360X	Nonsense	3.64	0.00	0.26	0.57	0.041
29	Ex11	c.1092_1095delT	p.F365fs407X	Deletion	1.82	0.00	0.51	0.57	0.337
30	IVS11	IVS11 + 1G > A	p.340_392del	Splice-site	5.45	2.47	0.51	1.33	0.008
31	Ex12	c.1215G > T	p.K405N	Missense	1.82	0.00	0.00	0.19	0.104
32	Ex13	c.1231G > A	p.V411M	Missense	0.00	0.00	0.26	0.19	1.000
33	Ex14	c.1364G > T	p.R455L	Missense	3.64	0.00	0.00	0.38	0.008
34	Ex14	c.1381G > T*	p.E461X	Nonsense	1.82	0.00	0.00	0.19	0.104
35	Ex14	c.1399C > T	p.R467X	Nonsense	0.00	6.17	0.77	1.52	0.007
36	Ex14	c.1452 + 1G > A*	p.A438_K484del	Splice-site	0.00	0.00	0.26	0.19	1.000
37	Ex16	c.1622C > A	p.A541D	Missense	0.00	1.23	0.00	0.19	0.258
38	Ex16	c.1638_1660dup	p.A554fs570X	Duplication	16.36	7.41	7.65	8.52	0.089
39	Ex16	c.1706_1707delTA*	p.S331fsX363	Deletion	0.00	1.23	0.00	0.19	0.258
40	IVS16	IVS16ins3kb	p.A584fs585X	Insertion	20.00	16.05	7.40	10.04	0.002
41	Ex17	c. 1775A > C	p.Q592P	Missense	0.00	0.00	0.26	0.19	1.000
42		Unknown			1.82	0.00	1.28	1.14	0.478
		Total			100.00	100.00	100.00	100.00	

^*^Novel mutations. The *P* value is for the comparison of the relative frequencies among three different areas.

^※^Reported in the refs 29,41 and 42.

^▲^Identified in ref. 42.

**Table 3:** The *SLC25A13* ASVs detected by cDNA cloning analysis in patients C0360 and C0388.

Patients	No.	ASVs	Annotations	Clones
C0360	1	*r.*213_468del	Exon 4, 5 skipping	15
	2	*r.*213_468del; *r.*616_754del	Exon 4, 5, 7 skipping	1
	3	*r.*213_468del; *r*.1453_1591del	Exon 4, 5, 15 skipping	3
	4	*r.*70_468del	Exon 3, 4, 5 skipping	1
	5	*r*.69_70ins69 + 12147_69 + 12282; *r.*213_468del	Exon 4, 5 skipping with partial intron 2 retention	1
	6	*r.*328_468del	Exon 5 skipping	2
	7	*r.*328_468del; *r.*755_848del	Exon 5, 8 skipping	1
			Total	24
C0388	1	*r.*213_328del, *r*.851_854del	Exon 4 skipping, *r*.851_854del	2
	2	*r.*213_328del, *r*.851_854del, *r*.1230_1231ins1230 + 1323_1230 + 1346	Exon 4 skipping, *r.*851_854del with partial intron 12 retention	1
	3	*r.*213_328del, *r*.616_ 754del, *r*.851_854del	Exon 4, 7 skipping, *r.*851_854del	1
	4	*r.*213_328del, *r*.616_ 848del, *r*.851_854del, *r*.993_1018del	Exon 4, 7, 8 skipping, *r*.851_854del with partial exon 10 deletion	1
	5	*r.*213_328del, *r.*755_848del, *r*.851_854del	Exon 4, 8 skipping, *r*.851_854del	3
	6	*r*.616_625del, *r.*755_848del, *r*.851_854del	Exon 8 skipping, *r*.851_854del with partial exon 7 deletion	1
	7	*r.*213_328del, *r*.851_854del, *r*.1312_1452del	Exon 4, 14 skipping, *r*.851_854del	3
	8	*r.*213_328del, *r.*755_848del, *r*.851_854del, *r*.1312_1452del	Exon4, 8, 14 skipping, *r*.851_854del	4
	9	*r.*213_468del, *r.*755_848del, *r*.851_854del, *r.*1312_1452del	Exon 4, 5, 8, 14 skipping, *r*.851_854del	1
	10	*r.*213_328del, *r.*755_933del, *r*.1312_1452del	Exon4, 8,,9, 14 skipping	3
	11	*r.*213_328del, *r*.851_854del, *r*.1312_1591del, *r*.851_854del	Exon 4, 8, 14, 15 skipping, *r*.851_854del	1
			Total	21

ASVs: alternative splicing variants. In patient C0360, the ASVs from the maternal *SLC25A13* allele were not detected due to the mutation IVS16ins3kb, and thus all 24 detected ASVs in this patient had paternal origin, all featuring exon 5 skipping. In patient C0388, all 21 ASVs were from the maternal *SLC25A13* allele harboring mutation [c.851_854del4; c.1452 + 1G > A], while those from the paternally-inherited allele with obscure mutation were not detected.

**Table 4:** Comparison of the *SLC25A13* genotypes of the CD patients from different areas in China.

NO.	Genotype	Relative frequency (%)				*P*
		North (n = 23)	Border (n = 35)	South (n = 187)	China (n = 245)	
01	c.851_854del4/c.851_854del4	4.35	28.57	49.20	42.04	0.000
02	c.1638_1660dup/c.1638_1660dup	4.35	2.86	1.60	2.04	0.339
03	IVS6 + 5G > A/IVS6 + 5G > A	0.00	0.00	0.53	0.41	1.000
04	IVS16ins3kb/IVS16ins3kb	8.70	2.86	1.60	2.45	0.095
05	c.851_854del4/c.1638_1660dup	8.70	0.00	8.02	6.94	0.190
06	c.851_854del4/IVS6 + 5G > A	0.00	5.71	9.09	7.76	0.374
07	c.851_854del4/IVS16ins3kb	21.74	14.29	8.56	10.61	0.083
08	c.851_854del4/c.103A > G (p.M35V)	0.00	0.00	0.53	0.41	1.000
09	c.851_854del4/IVS4ins6kb	4.35	0.00	1.60	1.63	0.407
10	c.851_854del4/c.329−1687_c.468 + 3865del	0.00	0.00	0.53	0.41	1.000
11	c.851_854del4/c.550C > T (p.R184X)	0.00	0.00	0.53	0.41	1.000
12	c.851_854del4/c.775C > T (p.Q259X)	0.00	2.86	0.00	0.41	0.237
13	c.851_854del4/c.847G > T (p.G283X)	0.00	0.00	0.53	0.41	1.000
14	c.851_854del4/c.955G > A (p.R319X)	4.35	0.00	0.00	0.41	0.094
15	c.851_854del4/c.998G > A (p.G333D)	0.00	0.00	0.53	0.41	1.000
16	c.851_854del4/c.1019_1177 + 893del	4.35	0.00	0.00	0.41	0.094
17	c.851_854del4/c.1048G > A (p.D350N)	0.00	0.00	1.07	0.82	1.000
18	IVS6 + 5G > A/c.1064G > A (p.R355Q)	0.00	0.00	1.07	0.82	1.000
19	c.851_854del4/c.1078C > T (p.R360X)	0.00	0.00	0.53	0.41	1.000
20	c.851_854del4/c.1092_1095delT	0.00	0.00	0.53	0.41	1.000
21	c.851_854del4/c.1215G > T (p.K405N)	4.35	0.00	0.00	0.41	0.0.94
22	c.851_854del4/c.1231G > A (p.V411M)	0.00	0.00	0.53	0.41	1.000
23	c.851_854del4/c.1399C > T (p.R467X)	0.00	8.57	1.07	2.04	0.032
24	c.851_854del4/c.1775A > C (p.Q592P)	0.00	0.00	0.53	0.41	1.000
25	c.1638_1660dup/IVS6 + 5G > A	0.00	2.86	2.14	2.04	0.744
26	c.1638_1660dup/IVS16ins3kb	4.35	2.86	1.60	2.04	0.339
27	c.1638_1660dup/c.550C > T (p.R184X)	0.00	2.86	0.00	0.41	0.238
28	c.1638_1660dup/c.265delG	0.00	0.00	0.53	0.41	1.000
29	c.1638_1660dup/c.1063C > G (p.R355G)	0.00	0.00	0.53	0.41	1.000
30	c.1638_1660dup/IVS11 + 1G > A	4.35	0.00	0.00	0.41	0.094
31	c.1638_1660dup/c.1364G > T (p.R455L)	4.35	0.00	0.00	0.41	0.094
32	c.1638_1660dup/c.1381G > T (p.E461X)	4.35	0.00	0.00	0.41	0.094
33	IVS6 + 5G > A/IVS16ins3kb	0.00	2.86	1.07	1.22	0.557
34	IVS6 + 5G > A/c.955G > A (p.R319X)	4.35	0.00	1.07	1.22	0.306
35	IVS6 + 5G > A/IVS11 + 1G > A	0.00	2.86	0.00	0.41	0.237
36	IVS6 + 5G > A/c.1399C > T (p.R467X)	0.00	0.00	0.53	0.41	1.000
37	IVS6 + 5G > A/IVS4ins6kb	0.00	2.86	0.00	0.41	0.237
38	IVS16ins3kb/[c.329−154_c.468 + 2352del2646; c.468 + 2392_c.468 + 2393ins23]	0.00	2.86	0.00	0.41	0.237
39	IVS16ins3kb/IVS11 + 1G > A	4.35	0.00	0.00	0.41	0.094
40	IVS16ins3kb/c.495delA	0.00	2.86	0.00	0.41	0.237
41	IVS16ins3kb/c.1048G > A (p.D350N)	0.00	0.00	1.07	0.82	1.000
42	IVS16ins3kb/c.1063C > G (p.R355G)	0.00	0.00	0.53	0.41	1.000
43	IVS16ins3kb/c.493C > T (p.Q165X)	0.00	2.86	0.00	0.41	0.237
44	g.2T > C/r.16_212dup	0.00	0.00	0.53	0.41	1.000
45	c.2T > C/c.790G > A (p.V264I)	0.00	0.00	0.53	0.41	1.000
46	IVS11 + 1G > A/c.845_c.848 + 1delG	0.00	0.00	0.53	0.41	1.000
47	IVS11 + 1G > A/c.1078C > T (p.R360X)	4.35	0.00	0.00	0.41	0.094
48	c.550C > T (p.R184X)/IVS4ins6kb	0.00	0.00	0.53	0.41	1.000
49	c.1399C > T (p.R467X)/IVS4ins6kb	0.00	2.86	0.00	0.41	0.237
50	1092-1095delT/c.754G > A	0.00	0.00	0.53	0.41	1.000
51	c.475C > T (p.Q159X)/c.1399C > T (p.R467X)	0.00	2.86	0.00	0.41	0.237
52	IVS7-1G > C/c.1364G > T (p.R455L)	4.35	0.00	0.00	0.41	0.094
53	c.775C > T (p.Q259X)/c.72T > A (p.Y24X)	0.00	2.86	0.00	0.41	0.237
	Total	100.00	100.00	100.00	100.00	

The *P* value is for the comparison of the relative frequencies among the three different areas.
