# Supplementary material for: Molecular diagnosis of pediatric patients with citrin deficiency in China: SLC25A13 mutation spectrum and the geographic distribution
Source: Sci Rep. 2016 Jul 11;6:29732. doi: 10.1038/srep29732 (PMC4942605; doi:10.1038/srep29732)
Supplement: Supplementary Information [file srep29732-s1.pdf]

Molecular diagnosis of pediatric patients with citrin deficiency in China: *SLC25A13* mutation spectrum and the geographic distribution

Wei-Xia Lin, Han-Shi Zeng, Zhan-Hui Zhang, Man Mao, Qi-Qi Zheng, Shu-Tao Zhao, Ying Cheng, Feng-Ping Chen, Wang-Rong Wen & Yuan-Zong Song

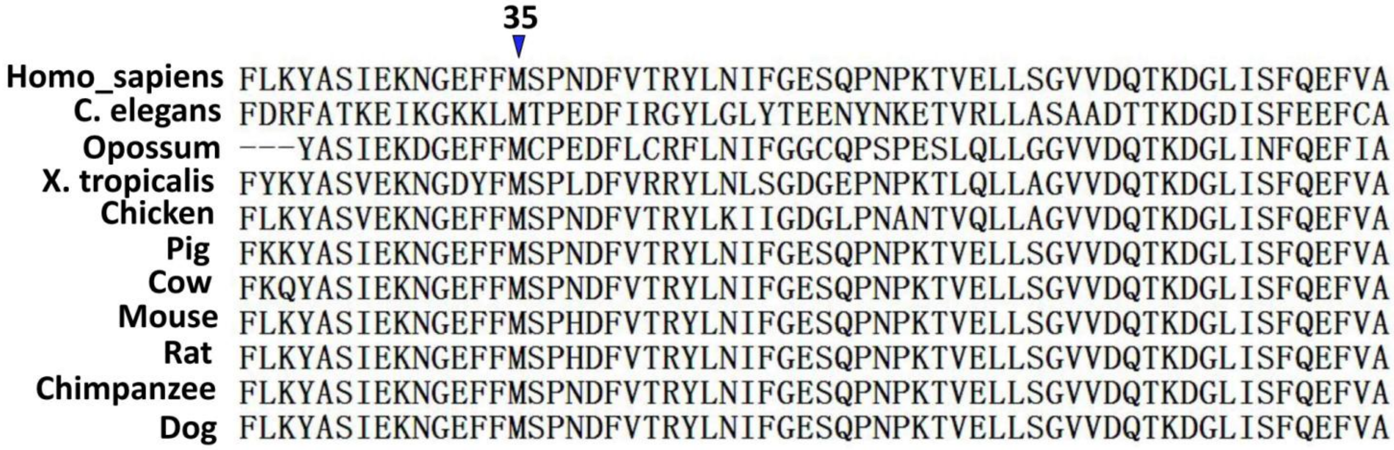

Supplemental Information 1. Alignment analysis of the homologous proteins. The mutation p.M35V affected an amino acid highly conserved in all 11 homologous proteins from 11 eukaryotic species, including human (Homo sapiens), chimpanzee, dog, pig, cow, rat, mouse, chicken, xenopus tropicalis (X. tropicalis), opossum and caenorhahditis elegans (C. elegans).
